# Attention Network Dysfunctions in Lewy Body Dementia and Alzheimer’s Disease

**DOI:** 10.3390/jcm13226691

**Published:** 2024-11-07

**Authors:** Yujing Huang, Ruth Cromarty, Lina Jia, Ying Han, John O’Brien, John-Paul Taylor, Li Su

**Affiliations:** 1 Department of Psychiatry, University of Cambridge, Cambridge CB22QQ, UK; huangyujing14@westlake.edu.cn (Y.H.);; 2 Zhejiang Key Laboratory of Multi-Omics in Infection and Immunity, Center for Infectious Disease Research, School of Medicine, Westlake University, Xihu District, Hangzhou 310024, China; 3 Research Center for Industries of the Future, School of Life Sciences, Westlake University, Xihu District, Hangzhou 310024, China; 4 Institute of Neuroscience, Newcastle University, Campus for Ageing and Vitality, Newcastle upon Tyne NE17RU, UKjohn-paul.taylor@ncl.ac.uk (J.-P.T.); 5 Beijing Anding Hospital, Capital Medical University, Beijing 100088, China; 6 Beijing Xuanwu Hospital, Capital Medical University, Beijing 100088, China; 7 Department of Neuroscience, Neuroscience Institute, Insigneo Institute for In Silico Medicine, University of Sheffield, Sheffield S102TN, UK

**Keywords:** attention dysfunction, source-level electroencephalograph, functional magnetic resonance imaging, Lewy body dementia, Alzheimer’s disease, ANT

## Abstract

**Background:** Attention deficits are notable in Lewy body dementia (LBD) and in Alzheimer’s disease (AD). In this study, we combined functional magnetic resonance imaging (fMRI) and electroencephalograph (EEG) to detect neural correlates of attention dysfunctions in LBD and AD. **Methods:** We recruited 33 patients with LBD, 15 patients with AD and 19 elderly healthy controls. The participants performed the modified Attention Network Task (ANT) to investigate the attention dysfunctions. **Results:** We found that LBD had alerting attention deficits and AD showed apparent orienting attention dysfunctions, while LBD and AD maintained relatively normal executive/conflict attention. Based on source-level EEG analyses, LBD had frontal-central deficits for alerting attention while AD showed inferior frontal and precentral impairments for orienting attention. In addition, the insular and inferior frontal areas were hyper-activated in LBD and AD for executive/conflict attention. Apart from these areas, LBD showed activity in the complementary temporal-central-occipital network for the modified ANT task. Furthermore, the oscillational sources for the ANT effects indicated that the alpha and theta bands were partly impaired in dementia patients. **Conclusions:** In summary, using source-localised EEG, we found that attention dysfunctions in LBD and AD engaged different neural networks.

## 1. Introduction

The concept of α-synucleinpathies with Lewy bodies, also termed Lewy Body Dementia (LBD), consists of dementia with Lewy Bodies (DLB), Parkinson’s disease (PD), and Parkinson’s disease dementia (PDD). Among these, DLB accounts for an incidence of 3.2–7.1% and a prevalence of 0.3–24.4% among all dementia cases [1]. The incidence and prevalence of LBD patients is recognized as second after Alzheimer’s disease (AD) [2]. The core clinical features in LBD include recurrent visual hallucinations, sleep and movement disorders, poor regulation of body functions and cognitive problems [3]. Due to overlap symptoms between LBD and AD, misdiagnosis is common.

Recent studies have suggested that distinct patterns of attention dysfunction may play a role in differentiating LBD from AD [4,5,6]. LBD patients frequently have significant disturbances in attention (e.g., alertness or arousal), which is primarily manifested clinically as cognitive fluctuation [7,8,9]. For example, patients with LBD appear ‘blank’, ‘vague’ or ‘unresponsive’ [9,10]. Likewise, impaired attentional arousal in LBD has been emphasized as the form of intermittent drowsiness and lethargy [11] or transient confusion on waking [9]. However, AD patients are pronounced regarding impairments of attentional orientation to external stimuli [12,13,14,15]. Patients with AD have profound visuospatial deficits [16], oculomotor apraxia and optic ataxia [17]. In addition, AD is associated with signs of spatial neglect or simultanagnosia, which is not being able to perceive items simultaneously, and shows impairments in spatial restriction of attention [18].

Until now, knowledge about the neural correlations of attention impairments in AD and LBD was still controversial. It was demonstrated that disease progression in LBD was associated with the ventral attention network (VAN), dorsal attention network (DAN) [19,20] or salience network dysfunctions [19,21,22]. For example, it was observed the thalamic atrophy in the salience network among DLB patients was implicated in the impaired maintenance of attention [19]. Also, there was an association between cognitive fluctuation and thalamic perfusion in DLB patients [22]. Furthermore, resting-state functional magnetic resonance imaging (fMRI) showed that the functional connectivity of the ventral and dorsal attention network was reduced in LBD [23]. However, the impairments in the dorsal attentional network in the early stage of AD was also observed. Specifically, the early stage of AD showed decreased regional homogeneity in the right middle temporal gyrus (MTG), and increased functional connectivity in the left inferior parietal lobule (IPL), which were known as parts of DAN [24]. Additionally, the attention-task-related fMRI indicated that tau and ptau in AD patients were highly colocalized with the attentional or executive control tissues (e.g., the right dorsal lateral prefrontal cortex, anterior cingulate cortex, and lateral parietal regions) [25]. Furthermore, frontal eye lobe atrophy in the dorsal attention network has been implicated for attentional dysfunction in AD [16,17]. AD patients had hyperconnectivity between the posterior part of the default mode network and the dorsal attention network [25]. An increasing number of Electroencephalography (EEG) research assessed the progressive cognitive decline in LBD or AD. For instance, Aoki et al. found that decreased occipital activities correlated with attention/concentration dysfunctions in DLB [26]. Although these findings from fMRI or EEG alone are important, multi-model imaging tools with both temporal and spatial resolution will be more insightful for detecting neural activity alterations in LBD or AD.

The aim of this study was to examine the underlying attention neural network dysfunctions in LBD and AD. The modified Attention Network Task (ANT) [27,28,29,30,31], convenient for measuring individual attention-deficit disorders, has been used to investigate the attentional dysfunction for AD and LBD [32,33]. It detects the extra time to resolve conflict between a central target and surrounding flankers that signal the same or an opposite response [33,34]. Cues prior to the target are commonly adopted by scholars to measure the efficiency of alerting and orienting attention. It has been demonstrated that the alerting, orienting and executive attention components have different dominant neuromodulators and uncorrelated networks [28]. We separately measured neural activities via EEG and fMRI for LBD and AD patients while performing the ANT task. In order to localise the scalp EEG to its cortical sources, spatial priors from fMRI have been used. We hypothesized that patients with LBD would have impairments in alerting attention while AD patients would display deficits in orienting attention. The activities in VAN, DAN or the thalamus in the salience network may be involved in revealing the differences between AD and LBD pathologies.

## 2. Materials and Methods

### 2.1. Participants

Sixty-seven elderly subjects (15 AD, 18 DLB, 15 PDD and 19 elderly healthy controls) participated in this study. All patients with dementia were recruited from referrals by local old age psychiatry and neurology services, or from the local community-dwelling population. The diagnosis of probable LBD or probable AD was conducted by two experienced psychiatrists independently. The criteria for the diagnosis of LBD were based on the International Consensus Guidelines [35]. The National Institute on Aging-Alzheimer’s Association [36] was used for the diagnosis of AD. Healthy controls (HC) were similarly aged and cognitively normal (Mini-Mental State Examination, MMSE ≥ 28) individuals. They were recruited from friends or spouses of patients with no history of significant neurological or psychiatric diseases. All participants underwent a standard battery of tests, including MMSE, Cambridge Cognitive Examination (CAMCOG), Neuropsychiatric Inventory (NPI), Unified Parkinson’s Disease Rating Scale (UPDRS), and Clinical Assessment of Fluctuation (CAF) [37]. People with alcohol or substance abuse, severe visual impairment, cerebral small vessel disease, contra-indications for magnetic resonance imaging, focal brain lesions on brain imaging or other severe medical illnesses were excluded from this study. The study was approved by the local ethics committee and written consent forms were obtained from each subject before their participation according to the Declaration of Helsinki. All patients had taken their usual anti-Parkinsonian medications during the period of this study.

### 2.2. Experimental Procedure

**Figure 1:**
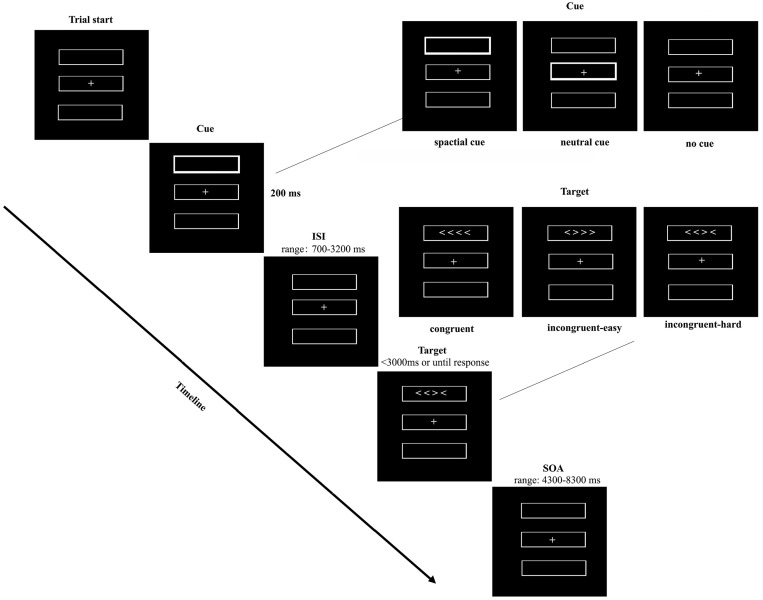
ANT paradigm. The figure presents three cue conditions (no cue, neutral cue, spatial cue) and three target conditions (congruent, incongruent-easy, incongruent-hard). The display consisted of a central fixation cross and three vertical-arrayed white boxes against a black background. One of three possible cues (no cue, neutral cue, spatial cue) was presented for 200 ms. In the neutral cue condition, the central box flashed. In the spatial cue condition, one of the boxes either above or below the central fixation flashed to show the participant the position of the subsequent target at a 100% ratio. Target stimuli consisted of four horizontal-arrayed white arrowheads pointing leftward or rightward. The target was presented randomly in the box above or below the fixation cross. The arrowheads either all pointed in the same direction (congruent), one arrowhead at the end of row pointing in the opposite direction (incongruent-easy), or one of the middle two arrowheads pointing in the opposite direction (incongruent-hard). The task required the participants to identify the direction in which the majority of arrowheads were facing. The target remained on screen until the participant responded by squeezing an air pressure bulb as quickly and accurately as possible, or had elapsed until 3000 ms.

As shown in Figure 1, each trial in the ANT task started with a fixation cross in the centre of the screen, followed by three boxes vertically arranged on the screen. Then, one of three cue conditions (i.e., no cue, neutral cue, spatial cue) was presented for 200 ms. A neutral cue was presented at the central position. A spatial cue was presented either above or below the central fixation to indicate the appearance of a subsequent target position. In the no cue condition, the screen remained unchanged. Then, a target, comprised of four horizontally arranged arrowheads (horizontal visual angle of 0.48 degrees), was presented in a box. The inter-stimulus interval (ISI) between the cue and the target was randomly sampled from an exponential distribution with the following values: 700, 770, 850, 960, 1080, 1240, 1430, 1660, 1940, 2300, 2700, 3200 ms (mean duration = 1800 ms). The target stimuli had three conditions: congruent, incongruent-easy and incongruent-hard. The congruent target means that all arrowheads were pointing in the same direction (left or right). The incongruent-easy target means that one incongruent arrowhead always appeared at the end of the row. The incongruent-hard target means that one incongruent arrowhead appeared in the middle of the row, making it harder to be ignored. The participants were required to identify the direction of the majority of the arrowheads as quickly and accurately as possible by squeezing the air pressure bulb in the hand. The target remained on screen until the participants responded or the time elapsed was 3000 ms. The stimulus-onset asynchrony (SOA) between the target and the next trial cue was randomly selected from one of the following values: 4300, 4500, 4750, 5000, 5350, 5700, 6100, 6400, 6800, 7200, 7700, 8300 ms (mean duration = 6000 ms). Each SOA occurred 3 times for each run in a random order. During each run, the nine conditions (3 cues × 3 targets conditions) were presented in a counterbalanced pseudorandom order. There were 12 trials for each cue, leading to 18 congruent trials, 9 incongruent-easy trials and 9 incongruent-hard trials in each run. Participants completed between 3 and 14 runs of the task (median = 8). Each participant performed the ANT task during a fMRI scan and subsequently repeated the same task during an EEG recording on another day. Participants practised the task before starting the actual experiment.

### 2.3. Behavioural Data Analysis

Behavioural data were analysed using SPSS v20.0. Firstly, the between-group one-way analysis of variance (ANOVA) was applied to examine the accuracy or RT per cue condition (i.e., no cue, neutral cue and spatial cue, respectively). Then, another between-group one-way ANOVA was applied to analyse the accuracy or RT per target condition (i.e., congruent, incongruent-easy and incongruent-hard respectively). The RT of incorrect trials were excluded from the RT analyses. Secondly, we defined four attention components in the modified ANT task: (1) alerting attention: the neutral cue trials minus no cue trials for mean RT or accuracy, respectively; (2) orienting attention: the spatial cue trials minus neutral cue trials for mean RT or accuracy, respectively; (3) executive attention: all incongruent trials (easy and hard) minus congruent trials for mean RT or accuracy, respectively; (4) conflict attention: the incongruent-hard trials minus incongruent-easy trials for mean RT or accuracy, respectively. Then, the within-group one-sample T-tests were performed to analyse the magnitudes of attention components per subject group for each respective ANT attention component. Thirdly, between-group one-way ANOVA was separately applied to compare the magnitudes per attention component.

### 2.4. fMRI Data Acquisition and Analysis

In line with our previous studies [33,34], participants were scanned with body coil transmission and eight-channel head coil receiver using a 3T MR scanner (Achieva scanner, Philips Medical System, the Netherlands). Each participant had a magnetization prepared rapid gradient echo (MPRAGE) scan to acquire individual structural brain images. Then, we collected fMRI images with a gradient-echo (GE) echo planar imaging (EPI) sequence while participants were performing the ANT task. We used SPM12 version r7487 (Welcome Trust Centre for Neuroimaging, London, UK) and MATLAB version R2018b (9.5) (Mathworks, Natick, MA, USA) software for fMRI image pre-processing. Subsequently, a general linear model (GLM) was used at first-level analyses to generate the images per cue condition and images per target condition for each participant. After that, we calculated the fMRI images per ANT attention component (i.e., alerting, orienting, conflict and executive attention) for each participant, which were defined in the same way as the behavioural analyses. Next, the second-level mean fMRI images per ANT attention component were acquired for the AD, LBD and HC groups. Thus, the fMRI results (voxel-wise threshold *p* < 0.001) per ANT effect per subject group were used as spatial priors for subsequent EEG source localisation analyses.

### 2.5. EEG Data Acquisition and Analysis

After the fMRI scan on a different day, participants repeated the ANT task while EEG data were recorded. A 128-Ag/AgCl channel system with an online sampling rate of 1000 Hz was used. Electrode resistance was kept below 5 kΩ during EEG recording. The raw EEG data were pre-processed using SPM12. Firstly, the continuous EEG data were down-sampled to 250 Hz and corrected by applying two-pass butterworth filters, initially a high-pass filter of 1 Hz and then a low-pass filter of 100 Hz with zero phase shift. The 50 Hz noise was removed using a butterworth filter with a 3 dB pass-band ripple and 20 dB attenuation in the stop-band. Bad channels were marked with a threshold of 0.02. We excluded flat trials with a threshold of 0.05 μV and a sequence length of 100 ms. The threshold channel method was used to remove other EEG artefacts with a threshold of 1200 μV and an excision window of 200 ms. Then, EEG data were off-line re-referenced to the average of all electrodes.

Secondly, after cleaning the artefacts, EEG time series were time-locked to the onset of the cue (cue-locked) with a time window between 100 ms pre-stimulus and 200 ms post-stimulus onset. Similarly, EEG brain potentials were time-locked to the onset of the target (target-locked) with a time window between 100 ms pre-stimulus and 700 ms post-stimulus onset. The cue-locked and target-locked epochs were baseline-corrected relative to the pre-stimulus period. For each participant, averaged cue-locked epochs were calculated per cue condition (i.e., no cue, neutral cue and spatial cue). Similarly, averaged target-locked epochs were obtained per target condition (i.e., incongruent-hard target, incongruent-easy target and congruent target) for each participant. The ANT attention components were calculated for the averaged EEG potentials per participant using the same definitions as the behavioural and fMRI data. The cleaned sensor-level EEG data for ANT attention components were used for further source localisation analyses.

Thirdly, we estimated source-level EEG ANT effects based on the source reconstruction methods in SPM12. Individual anatomical images of each subject were segmented and spatially normalized to the Montreal Neurological Institute (MNI) template. Then, we applied the 3-Shell Spherical model for EEG forward computation and obtained the ‘lead-field’. The Multiple Sparse Priors (MSP) was optimized by the Greedy Search (GS) method for the inverse problem. We inverted the cleaned sensor-level EEG data per ANT attention component with the spatial priors (i.e., group-level fMRI images of corresponding attention components) after eight iterations of cortical smoothing. The EEG source-level time window per ANT attention component was consistent with sensor-level epoch time window. Next, five EEG frequency bands for the inversion were selected for subsequent analyses: overall (1–100 Hz), theta (4–7 Hz), alpha (8–13 Hz), beta (14–30 Hz) and gamma (31–100 Hz).

### 2.6. Statistical Analysis

For demographic and behavioural data (RT or accuracy), within-group one-sample T-tests and between-group one-way ANOVA were used for within-group comparisons and between-group comparisons, respectively. If the tests of homogeneity of variances between groups were significant (*p* < 0.05, two-tailed), Tamhane’s T2 was used for post-hoc comparisons; otherwise, we used Tukey’s tests for post-hoc comparisons. Gender, handedness and education analyses were compared via chi-square method.

For EEG, the source priors from fMRI analysis were obtained by performing voxel-wise one-sample *t*-tests with the threshold *p* < 0.001 (two-tailed) using SPM12. After the pre-processing steps, the within-group one sample *t*-tests were used to analyse grand mean EEG source-level images per attention component (i.e., alerting, orienting, executive and conflict) per respective subject group at each frequency band. Then, the between-group ANOVA was separately used to compare the magnitudes per ANT attention component using the source-level EEG images at each frequency band.

As regard to the oscillatory effects, the overall source-level EEG results at 1–100 Hz were from within-group one-sample *t*-tests (uncorrected *p* < 0.001, two-tailed) and the between-group one-way ANOVA with FWE corrected *p* < 0.05 (two-tailed). The oscillatory EEG results for alert, orient, conflict and executive attention were between-group one-way ANOVA (FWE corrected *p* < 0.05, two-tailed)

## 3. Results

### 3.1. Demographics

As shown in Table 1, there were no statistical differences in age, gender, education and handedness for the recruited participants [Age: F_(2,63)_ = 0.17; Gender: χ^2^_(2)_ = 3.06; Handedness: χ^2^_(4)_ = 4.06; Education: χ^2^_(6)_ = 6.13, all *p* > 0.05]. Here, Chi-square test was conducted to analyse the years of education, which was classified into four levels (i.e., level A: less than 9 years; level B: 9–11 years; level C: 12–13 years; level D: more than 14 years). Overall poorer cognitive functions were observed in dementia groups compared with HC [CAMCOG: F_(2,63)_ = 31.69; MMSE: F_(2,63)_ = 31.35, both *p* < 0.001]. In addition, the core features of LBD were significantly more present compared to AD or HC [UPDRS: F_(2,63)_ = 81.61; NPI hallucination: T_(32.61)_ = −4.28; CAF: T_(43.26)_ = −4.54, all *p* < 0.001].

### 3.2. Behavioural Results

Behavioural results are summarized in Table 2. Consistent with our previous study [33] patients with dementia (either LBD or AD) responded slower and less accurately than HC for ‘no cue’ [RT: F_(2,64)_ = 20.12; Accuracy: F_(2,64)_ = 10.11], ‘neutral cue’ [RT: F_(2,64)_ = 21.16; Accuracy: F_(2,64)_ = 7.54] and ‘spatial cue’ [RT: F_(2,64)_ = 18.79; Accuracy: F_(2,64)_ = 8.48] conditions, respectively (all *p* < 0.001). Also, HC had shorter reaction time and higher accuracy than patients with dementia (either LBD or AD) for ‘congruent target’ [RT: F_(2,64)_ = 20.82, *p* < 0.001; Accuracy: F_(2,64)_ = 4.10, *p* < 0.05], ‘incongruent-easy target’ [RT: F_(2,64)_ = 15.34; Accuracy: F_(2,64)_ = 8.41, both *p* < 0.001] and ‘incongruent-hard target’ [RT: F_(2,64)_ = 20.96; Accuracy: F_(2,64)_ = 10.51, both *p* < 0.001].

Using the within-group one-sample T-tests, we found that AD patients had preserved RT alert (RT: mean = −41.11, S.E. = 13.71, *p* < 0.01), conflict (RT: mean = 350.08, S.E. = 68.47, *p* < 0.001) and executive (RT: mean = 578.55, S.E. = 55.91, *p* < 0.001) attention, but impaired orienting attention (RT: mean = −64.99, S.E. = 32.90, *p* > 0.05). By contrast, LBD patients did not show RT alert (RT: mean = −17.20, S.E. = 16.68, *p* > 0.05) attention but relatively normal orient (RT: mean = −71.72, S.E. = 18.06, *p* < 0.001), conflict (RT: mean = 249.73, S.E. = 51.10, *p* < 0.001) and executive attention (RT: mean = 586.19, S.E. = 41.26, *p* < 0.001).

However, the alerting attention in accuracy was not apparent for HC, AD and LBD (Accuracy: HC mean = 0.05, S.E. = 0.61; AD mean = 1.33, S.E. = 1.19; LBD: mean = 0.88, S.E. = 1.01; all *p* > 0.05). There were impairments of orienting attention in accuracy for patients with dementia (either LBD or AD) compared to HC (Accuracy: HC mean = 0.79, S.E. = 0.36, *p* < 0.05; AD mean = −1.53, S.E. = 1.27, *p* > 0.05; LBD mean = −0.48, S.E. = 0.65, *p* > 0.05). For conflict and executive effects, HC and dementia patients showed similar responses (conflict in Accuracy: HC mean = 1.23, S.E. = 0.78, *p* > 0.05; AD mean = −6.79, S.E. = 2.57, *p* < 0.05; LBD mean = −4.71, S.E. = 1.73, *p* < 0.05; executive in Accuracy: HC mean = −1.77, S.E. = 0.48, *p* < 0.01; AD mean = −8.66, S.E. = 2.39, *p* < 0.01; LBD mean = −11.75, S.E. = 2.27, *p* < 0.001).

When we compared the between-group attention magnitudes per ANT attention component of RT or accuracy, no significant magnitude differences in alert and orienting attentions were found (Alert: RT F = 1.46, Accuracy F = 0.31; Orient: RT F = 0.37, Accuracy F = 1.74, all post-hoc *p* > 0.05). However, there were significant lower RT conflict/executive magnitudes and higher accuracy conflict/executive magnitudes in HC than dementia groups (Conflict: RT F = 4.59, Accuracy F = 4.25, both *p* < 0.05; Executive: RT F = 8.83, *p* < 0.001, Accuracy F = 5.69, *p* < 0.01).

### 3.3. Source-Level EEG Results

#### 3.3.1. EEG Source-Level Results at 1–100 Hz for ANT Attention Components

**Figure 2:**
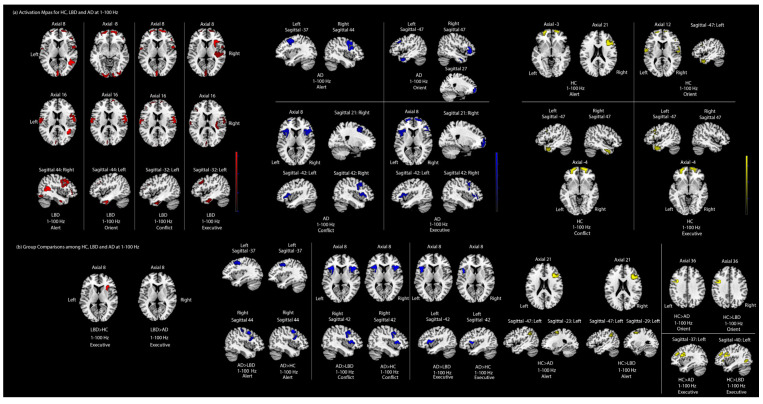
Overall source-level EEG results at 1–100 Hz. (**a**) Within-group one-sample *t*-tests for ANT attentional activation maps among HC, LBD and AD. The results are uncorrected *p* < 0.001. Yellow, blue and red indicated the significant areas for HC, AD and LBD, respectively. (**b**) Between-group one-way ANOVA results for group comparisons among HC, LBD and AD. All activated areas are family-wise error corrected *p* < 0.05. Yellow, blue and red represent the stronger activations in corresponding group comparisons. (Yellow: HC > AD or HC > LBD; Blue: AD > HC or AD > LBD; Red: LBD > HC or LBD > AD).

Using the within-group one-sample *t*-Test in Figure 2a, we found that healthy participants activated the bilateral superior frontal and right inferior frontal cortex for alerting attention at the overall frequency band (1–100 Hz). Although there were similar inferior frontal cortex activations among dementia patients (i.e., LBD or AD), middle frontal areas were more likely to be involved at the overall frequency band (1–100 Hz). Apart from these findings at the overall frequency band (1–100 Hz), dementia patients showed abnormal activations in bilateral precentral and postcentral areas for alerting attention. Additionally, LBD showed excessive activations at the temporal-to-occipital network (i.e., right middle temporal, right lingual areas and occipital areas) for alerting attention. The results of the between-group one-way ANOVA (see Figure 2b) further confirmed that AD had hyperactivations in the bilateral middle frontal areas, bilateral precentral areas, right postcentral areas as well as right inferior frontal areas, compared with LBD or HC for alerting attention. By contrast, HC had stronger activations in the left superior frontal areas, right inferior frontal areas and left inferior parietal areas than LBD or AD for alerting attention.

According to the within-group one-sample T-test results for orienting attention at the 1–100 Hz frequency band (Figure 2a), healthy adults involved a complex frontal-temporal network, including the left inferior/superior temporal and bilateral inferior/superior frontal cortex. We observed that dementia patients showed similar activations in the left inferior temporal, bilateral superior frontal cortex and right inferior frontal cortex. However, AD activated additional right temporal areas while LBD incorporated the complementary temporal-central-occipital network (i.e., bilateral inferior occipital areas, bilateral postcentral areas, right inferior temporal areas) for orienting attention. The between-group one-way ANOVA results (see Figure 2b) showed that LBD or AD were hypoactive in the left inferior frontal areas and left precentral areas for orienting attention compared to HC at an overall frequency band (1–100 Hz).

Although the behavioural conflict and executive attention results in AD were similar to that of LBD, the source-level EEG results provided insights into the distinct neural patterns of the central executive network among dementia patients. Specifically, the within-group one-sample T-test results (Figure 2a) showed that healthy participants showed activities in the bilateral inferior temporal and bilateral middle/superior frontal cortex for conflict and executive attention at 1–100 Hz. However, apart from the common involvement of middle/superior frontal areas, dementia patients had apparent inferior frontal activations for conflict/executive attention. In addition, AD impaired activities in inferior temporal areas for conflict/executive attention while LBD convened the neural activities in the central-to-occipital network (i.e., right precentral, bilateral postcentral, left inferior/superior parietal and middle occipital areas). Furthermore, insular is exclusively activated in LBD or AD groups compared to HC, especially for executive attention. According to the between-group one-way ANOVA results (Figure 2b), we found that AD had more hyperactivations than LBD or HC in bilateral inferior frontal areas and bilateral insula, coupled with increased activities in right middle frontal areas and right precentral areas for conflict attention. As for executive attention, we also detected upregulated activations in the left inferior frontal cortex and left insula in AD compared to LBD or HC. By contrast, HC had stronger activations in the middle/inferior frontal and precentral areas at the left hemisphere only for executive attention than AD or LBD. There were less hyperactivities (only the right insular for executive attention) in LBD compared to AD or HC at 1–100 Hz (All source-level EEG results above are summarized in Table 3).

#### 3.3.2. Alert and Orienting Attention Oscillation Effects

**Figure 3:**
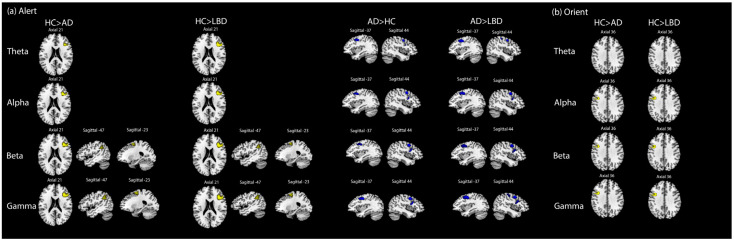
Oscillatory source-level EEG results for alert and orienting attention. (**a**) Between-group one-way ANOVA results for alert oscillation effects. (**b**) Between-group one-way ANOVA results for orient oscillation effects. The significant activation areas in HC and AD were marked as yellow and blue, respectively (family-wise error corrected *p* < 0.05). Four frequency bands were chosen for oscillation source-level analyses: theta band: 4–7 Hz, alpha band: 8–13 Hz, beta band: 14–30 Hz, gamma band: 31–100 Hz. The alerting attention of EEG source results was defined as ‘Alert = neutral cue-no cue’ with fMRI as priors. The orienting attention of EEG source results was defined as ‘Orient = spatial cue-neutral cue’ with fMRI as priors. The time window of alert or orienting attention was cue-evoked potentials from pre-stimulus 100 ms to post-stimulus 200 ms.

Figure 3a illustrated alerting attention oscillation dysfunction of dementia patients. The abnormal activities among dementia patients in right inferior frontal areas could be traced at all frequency bands. However, alerting attention impairments of LBD or AD in left superior frontal and left inferior parietal areas are more likely derived from beta and gamma bands. Compared to LBD or HC, AD’s hyperactivation in the right inferior frontal cortex may be rooted in alpha, beta and gamma bands, while AD’s abnormal activities in the bilateral middle frontal areas and bilateral precentral areas may be associated with four frequency bands.

The orientation oscillation effects were demonstrated in Figure 3b. We identified that the impairments of dementia patients in left inferior frontal and left precentral areas may be derived from alpha, beta and gamma bands. Although we observed distinct EEG source-level activation maps for LBD, AD and HC at 1–100 Hz (Figure 2a), there were no significant differences in orienting attention oscillation between LBD and AD.

#### 3.3.3. Conflict and Executive Attention Oscillation Effects

**Figure 4:**
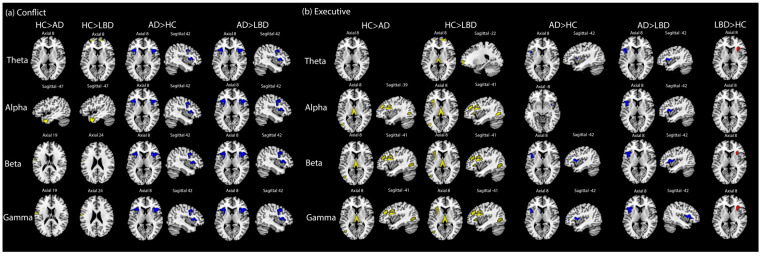
Oscillatory source-level EEG results for conflict/executive attention. (**a**) The conflict attention of EEG source results was defined as ‘Executive = incongruent target—congruent target’ with fMRI as priors. The significant activation areas in group comparisons were marked as different colors (family-wise error corrected *p* < 0.05) (Yellow: HC > LBD or HC > AD; Blue: AD > HC or AD > LBD; Red: LBD > HC or LBD > AD). Four frequency bands were chosen for oscillation source-level analyses: theta band: 4–7 Hz, alpha band: 8–13 Hz, beta band: 14–30 Hz, gamma band: 31–100 Hz. The time window of conflict attention was target-evoked potential from 100 ms pre-stimulus to 700 ms post-stimulus. (**b**) The executive attention was defined as ‘Conflict = incongruent hard target—incongruent easy target’. The time window of executive attention was target-evoked potential from 100 ms pre-stimulus to 700 ms post-stimulus. The symbols for colors and frequency bands were the same as the conflict attention.

As shown in Figure 4a, conflict attention oscillation dysfunction in dementia patients can be related to the alpha band in left temporal activations. Furthermore, compared to LBD or HC, AD’s impairments may be derived from all frequency bands in the bilateral insular, right inferior frontal areas and right precentral areas.

In regard to executive attention oscillation effects (see Figure 4b), there were apparently different sources among HC, LBD and AD, especially for inferior frontal areas. Specifically, healthy participants’ stronger activations in left inferior/middle frontal areas and left occipital areas relied on alpha, beta and gamma frequency bands. By contrast, the abnormal left inferior frontal activities in AD involved all frequency bands, particularly compared with LBD. As for LBD, dysfunctions in right inferior frontal areas and right insular areas can be observed at theta, beta and gamma bands compared to HC (All source-level EEG oscillatory results above are summarized in Table 4).

## 4. Discussions

In this study, we explored attention dysfunction (i.e., alert, orient, conflict and executive attention) in LBD and AD patients. Since attention processes involve large-scale brain network and are rapid in time, we have combined both high-temporal resolution provided by EEG and high-spatial resolution provided by fMRI, which facilitated the cortical source localisation of EEG signals. We analysed the oscillatory cortical activities by confining the separate time windows for cue-evoked and target-evoked stimuli in the modified ANT task. Initially, we observed typical demography and clinical features in LBD and AD patients. Behaviourally, LBD showed deficits in response speed for alerting attention while AD had dysfunctions in agility for the orienting attention. Both AD and LBD had stronger conflict and executive attention magnitudes than HC, indicating that impairments in central executive control abilities occur in all dementia types. Compared with accuracy, response speed is a better index of attention impairments in AD and LBD.

Neurologically, we found that alerting attention involved activation in temporal-to-occipital areas for LBD compared with AD. By contrast, AD showed more hyperactivities in middle frontal areas (i.e., theta, alpha, beta, gamma bands), precentral (i.e., theta, alpha, beta, gamma bands) and postcentral areas than LBD. Furthermore, healthy participants demonstrated more activities in superior frontal areas (i.e., beta and gamma bands) than all dementia patients. For orienting attention, AD had additional activations in right temporal areas while LBD involved the complementary temporal-central-occipital network. Both dementia groups showed impairments in the inferior frontal (i.e., alpha, beta, gamma bands) and precentral areas (i.e., alpha, beta, gamma bands) at the left hemisphere for orienting attention. For executive/conflict attention, the dementia groups were more activated at inferior frontal areas and insular areas (part of salience network) compared to healthy participants. Additionally, AD showed a lack of activations in the inferior temporal cortex (i.e., alpha band) for executive/conflict attention while LBD patients incorporate more activations in the central-to-occipital network. The oscillation effects of executive/conflict attention have indicated that although inferior frontal areas are involved among HC, AD and LBD, the oscillation sources are quite different (HC: alpha, beta, gamma bands; AD: theta, alpha, beta, gamma bands; LBD: theta, beta, gamma bands).

### 4.1. RT/Accuracy Discrimination Between AD and LBD in Attention Mechanisms

Existing research has shown more seriously impaired visuospatial functions in LBD than AD. For example, using a clinically validated cognitive test—ACE-R—we have shown that LBD and AD can be differentiated by memory/visuospatial functions [38]. Studies have also shown that LBD is poorer in processing speed in many clinical tests [39]. Caballero et al. have found slower responses in LBD patients than non-dementia patients in a simple choice reaction time task. Clinically, reaction time and accuracy in executive and attention tasks may have significant values in differential diagnoses for LBD and AD. As shown in our study, reaction time reductions in alerting attention or accuracy declines in orienting attention contribute to diagnostic processes or therapeutic strategies for LBD and AD.

### 4.2. Alerting Attention Dysfunctions in LBD

The present study confirmed that LBD and AD involved a severe generalized dysfunction and global attention function decline [40,41,42]. Attention deficits in dementia may be specific in the speed of responses [43] rather than accuracy. However, there are distinct characteristics in the alerting attention system between AD and LBD. LBD patients showed reduced reaction speed for alerting attention compared to AD. Previous research has demonstrated that noradrenergic mechanism impairments may reduce the alerting efficiency in LBD [44].

Neurologically, the dysfunction of frontal-central activation (i.e., superior frontal areas, middle frontal areas, precentral areas and postcentral areas) in LBD may be associated with inhibition in the frontal attention network, favoring the decoupling of the default mode network [45,46]. The frontal-central dysfunction was thought to be due to difficulties in unconstraining the default mode network, which may lead to hallucinations or attentional fluctuations—a core symptom of LBD [47]. Furthermore, another potential explanation for dysfunction of alerting attention in LBD may be related to the notion that LBD may involve a compensatory network in temporal-to-occipital areas for alerting attention [48,49]. Consistent with our findings, Schumacher et al. (2018) [50] emphasized the reduced connectivity in motor, frontal and temporal networks in LBD with relative sparing of the default mode network. Nicolas et al. [51] found that there was decreased clustering in bilateral temporal regions and decreased closeness centrality in middle central areas and the bilateral occipital lobe. Thus, LBD shows extensively disorganized modules, including in the dorsal attentional network and default-mode network.

### 4.3. Orienting Attention Impairments in AD

Until now, there were mixed findings on the impairments of orienting attention in AD and LBD. For example, some researchers found that AD patients had ‘anomalous’ responses to reaction time to invalid cues and costing reaction time for valid cues [52]. Other researchers observed that AD patients showed deficits in orient enhancement effects at short SOA (stimulus-onset asynchrony) for peripheral cues and at longer SOA (>500 ms) for central cues [53]. It has been discussed that orienting attention could be achieved by two simultaneously activated mechanisms [54]: reflexive attention, also termed as automatic or obligatory attention, is often evoked by highly salient cues or peripheral cues without affecting memory load; and voluntary attention, an effortful or controlled attention, can be initiated by central cues and abolished by memory load. The reflexive attention is mostly effective at short SOA [55,56], but when SOA is longer than 400 ms, peripheral or central cues involve the same voluntary mechanism as sustain attention [57]. In addition, previous literature has indicated that the orienting attention system consists of dorsal and ventral pathways [58,59,60]. The dorsal attention pathway contributes to the mediation of goal-directed tasks to select stimuli and evokes fronto-parietal areas, including intraparietal areas and the frontal eye field. By contrast, the ventral attention pathway is related to the temporal-parietal junction and ventral frontal areas. Attention shifts in spatially orienting cued tasks usually undergo complex processes of shifts (switching), engagement (focusing), maintenance (sustaining), and disengagement (reorienting) [61].

In our study, patients with AD may be more likely to have impairments in orienting attention processes than LBD. The central neutral cues and peripheral spatial cues evoked top-down voluntary attention (SOA > 900 ms), which may be associated with the neural resources in the frontoparietal attention pathway [62,63]. Furthermore, multi-abnormalities in AD might cause an inability to disengage eye movements and generate saccades to new targets from the current item [64]. The posterior atrophy in AD has shown hypometabolism of regions that are involved in the ventral and dorsal attention networks [65]. In addition, previous studies have claimed different roles of the bilateral frontal eye field in the attention system. The right-hemispheric dominance of the frontal eye field was suggested to modulate conscious visual perception and endogeneous (voluntary) attention processes [66]. However, the left frontal eye field was reported to fail in the modulation of conscious visual perception [67]. Studies on monkeys showed a correlation of exogeneous (reflexive) cover orienting in the activity of frontal eye field neurons during a pop-out visual search task [68]. In our study, the decreased inferior frontal activations and left precentral in AD may be involved in covert spatial attention [69,70,71] and saccade planning/production [72,73]. By contrast, although LBD had structural network atrophy in inferior frontal areas and precentral areas in the left hemisphere, LBD recruited a large-scale cluster of temporal-central-occipital network, which may play an important role in the execution of orienting attention.

### 4.4. Conflict/Executive Attention Dysfunctions in LBD and AD

The conflict/executive attention in the modified ANT task encompasses the underlying relevance to working memory and inhibition control [74]. Poor performances in incongruent stimuli indicated the inability to deal with resolution of conflict, which has been found to be linked to the dorsal anterior cingulated cortex atrophy in AD [75]. The impairments in executive function were also reported in other tasks for AD patients, such as reduced accuracy in the flanker task [76]. In a large cohort study, cluster analyses identified the predominantly executive impairments relatively early in the dementia progression of AD [60].

The executive dysfunctions in the current study fit well with dopaminergic system disorders, which are critical in maintaining executive function in patients with Lewy bodies [77,78,79,80]. A hallmark of LBD is the loss of dopaminergic neurons in the substantia nigra. Previous study has demonstrated a significant negative correlation between cognitive fluctuation severity and striatal dopamine transporter density in LBD [81]. In addition, atrophy in the anterior cingulated cortex may be a prodromal manifestation of LBD [82].

It has been demonstrated that the insular area (part of salience network) plays a key role in visual hallucination manifestation, which more markedly and pathologically appears in dementia patients than AD [83]. However, the roles of insular in conflict/executive attention of dementia patients may be multi-dimensional. There are at least three speculations about the roles of the insular area according to our knowledge. Firstly, some researchers reported the hyperactivation of the insular area in the salience network in patients with anxiety disorders [84] and schizophrenia [85]. It seems that insular activation may be related to emotional arousal or distress. Another potential speculation of the insular area may be further related to the salience of the subsequent targets, which is probably a compensatory in conflict/executive attention efficiency for dementia patients. Thirdly, the function of the insular area is also related to a switch between default model network and central executive network [86,87]. Consistent with the literature, our data also showed a specific involvement of the insular area in dementia patients for attention/executive attention.

### 4.5. Oscillatory Cortical Neural Network

Combining high-temporal resolution EEG and high-spatial resolution fMRI imaging tools, our study revealed multi-model oscillatory cortical networks that distinguish LBD from AD. We found that low-frequency bands, such as the theta and alpha band, were faintly discernible in alert and orienting attention for AD or LBD patients. In line with the previous study, dynamic oscillations of theta and alpha bands are closely related to the dorsal and ventral attention pathways [88]. In addition, it was investigated that patients with rapid eye movement sleep disorders, which is a core feature of LBD, showed dysfunction in low-frequency bands during wakefulness [89]. Furthermore, there was evidence that the theta burst mode and theta electroencephalogram activities were related to subsequent thalamocortical dysrhythmia [47].

### 4.6. Limitations

Although our findings in attentional component differences between AD and LBD is interesting, there are still some limitations. First of all, since the prevalence and incidence of LBD is not high (e.g., DLB: 2.4%−5.9% in individual services [90]), the sample size in our study is rather moderate. Secondly, the gender effect of dementia patients is not estimated in the current study; existing literature showed that LBD is more prevalent in males and is a rarer condition than AD [91]. As such, the sex ratio in two groups may be biased toward male patients. In addition, the study was done in the UK, so the majority of participants are Caucasians. Future studies should study the effect in other ethics groups and in females.

## 5. Conclusions

In conclusion, the current study investigated the dysfunction of alerting, orienting, conflict and executive attention in AD and LBD. According to the findings, there were alerting attention deficits in patients with LBD, which may be related to the possible frontal-central dysrhythmia compared to AD. In addition, the orienting attention system in LBD involved an additional stronger activation of the temporal-central-occipital network in order to evoke the orient efficiency than AD. However, AD with left inferior frontal and precentral cortex dysfunction had deficits in orienting attention, indicating ventral and dorsal network impairments. Lastly, the low-frequency oscillation may be deficits in LBD about the dysrhythmia or dorsal/ventral attention pathways.

## Figures and Tables

**Table 1:** Demographics and clinical scores.

	HC N = 19	AD N = 15	LBD ^a^ N = 32	Statistics	Post-Hoc Test
Age (Mean ± S.E.)	75.79 ± 1.26	74.47 ± 2.19	75.16 ± 1.07	F_(2,63)_ = 0.17	HC =AD = LBD ^b^
Education (Level A:B:C:D)	0:12:1:6	0:10:2:3	3:21:4:4	χ^2^_(6)_ = 6.13	HC = AD = LBD ^c^
Gender (female:male)	5:14	5:10	4:28	χ^2^_(2)_ = 3.06	HC = AD = LBD ^c^
Handedness (right:left:ambidextrous)	18:1:0	14:1:0	30:0:2	χ^2^_(4)_ = 4.06	HC = AD = LBD ^c^
CAMCOG (Mean ± S.E.)	96.89 ± 0.84	73.07 ± 2.73	75.22 ± 2.23	F_(2,63)_ = 31.69 ***	HC > AD = LBD ^d^
MMSE (Mean ± S.E.)	29.26 ± 0.18	21.86 ± 0.92	23.22 ± 0.63	F_(2,63)_ = 31.35 ***	HC > AD = LBD ^d^
UPDRS (Mean ± S.E.)	1.05 ± 0.33	2.07 ± 0.45	20.84 ± 1.52	F_(2,63)_ = 81.61 ***	HC = AD < LBD ^d^
NPI total (Mean ± S.E.)	-	8.67 ± 1.94	13.81 ± 1.80	T_(45)_ = −1.73	AD = LBD ^e^
NPI hallucination (Mean ± S.E.)	-	0.07 ± 0.06	1.84 ± 0.40	T_(32.61)_ = −4.28 ***	AD <<< LBD ^e^
CAF(Mean ± S.E.)	-	0.67 ± 0.43	4.88 ± 0.81	T_(43.26)_ = −4.54 ***	AD <<< LBD ^e^

‘Education for Level A, B, C and D’ means years of education less than 9 years (level A), 9–11 years (level B), 12–13 years (level C) and more than 14 years (level D). Total scores for CAMCOG, MMSE, UPDRS, CAF are illustrated; ^‘a’^ indicates that LBD consists of 14 PDD and 18 DLB patients, ‘^b^’, ‘^c^’, ‘^d^’ and ‘^e^’ indicate that the post-hoc method is Tukey, Pearson chi square, Tamhane’s and Independent-sample T tests, respectively; The symbols, ‘<<<’ and ‘***’ indicate *p* ≤ 0.001; the symbols of ‘>’ and ‘<’ indicate *p* ≤ 0.05, the symbol of ‘=’ for post-hoc tests indicates *p* > 0.05. The unit of education or age is ‘years’. The between-group one-way ANOVA was used for the statistical analyses for age, CAMCOG, MMSE and UPDRS, respectively. The scores of NPI total, NPI hallucination and CAF are based on independent-sample T tests. The analyses of education, gender and handedness were via chi-square tests. S.E. means standard errors.

**Table 2:** Reaction time and accuracy rate of ANT task during EEG.

	HC (n = 19) Mean (S.E.)	AD (n = 15) Mean (S.E.)	LBD ^a^ (n = 33) Mean (S.E.)	Between-Group ANOVA Comparison (df = 2, 64)	Between-Group Tamhane’s T2 Post-Hoc Comparison
**RT for correct trials (ms)**					
No cue	1064.23 (37.63)	1486.06 (112.17)	1751.87 (75.02)	F = 20.12 ***	HC < AD = LBD
Neutral cue	1011.30 (33.78)	1444.94 (109.30)	1734.66 (79.36)	F = 21.16 ***	HC < AD = LBD
Spatial cue	919.62 (30.70)	1379.95 (132.64)	1662.94 (83.29)	F = 18.79 ***	HC < AD = LBD
Congruent	766.13 (23.60)	1051.28 (90.12)	1325.69 (61.49)	F = 20.82 ***	HC < AD = LBD
Incongruent-easy	1066.23 (39.62)	1454.80 (117.00)	1787.02 (96.18)	F = 15.34 ***	HC < AD = LBD
Incongruent-hard	1162.79 (46.14)	1804.88 (155.17)	2036.75 (89.23)	F = 20.96 ***	HC < AD = LBD
**Accuracy rate (%)**					
No cue	97.72 (0.68)	85.72 (3.95)	79.96 (2.85)	F = 10.11 ***	HC > AD = LBD
Neutral cue	97.80 (0.44)	87.01 (4.37)	80.86 (3.16)	F = 7.54 ***	HC = AD; HC > LBD; AD = LBD
Spatial cue	98.61 (0.36)	85.39 (4.62)	80.38 (3.17)	F = 8.48 ***	HC > AD = LBD
Congruent	99.22 (0.34)	91.81 (4.40)	88.23 (2.61)	F = 4.10 *	HC = AD; HD > LBD; AD = LBD
Incongruent-easy	96.84 (0.66)	86.55 (4.10)	78.84 (3.25)	F = 8.41 ***	HC = AD; HD > LBD; AD = LBD
Incongruent-hard	98.07 (0.67)	79.76 (5.03)	74.13 (3.87)	F = 10.51 ***	HC > AD = LBD
**RT differences for correct trials (ms)**					
Alert	−52.92 (9.82) ***	−41.11 (13.71) **	−17.20 (16.68)	F = 1.46	HC = AD = LBD ^b^
Orient	−91.68 (9.29) ***	−64.99 (32.90)	−71.72 (18.06) ***	F = 0.37	HC = AD = LBD
Conflict	96.56 (24.87) ***	350.08 (68.47) ***	249.73 (51.10) ***	F = 4.59 *	HC < AD = LBD
Executive	348.38 (28.66) ***	578.55 (55.91) ***	586.19 (41.26) ***	F = 8.83 ***	HC < AD = LBD
**Accuracy rate difference (%)**					
Alert	0.05 (0.61)	1.33 (1.19)	0.88 (1.01)	F = 0.31	HC = AD = LBD ^b^
Orient	0.79 (0.36) *	−1.53 (1.27)	−0.48 (0.65)	F = 1.74	HC = AD = LBD
Conflict	1.23 (0.78)	−6.79 (2.57) *	−4.71 (1.73) *	F = 4.25 *	HC > AD = LBD
Executive	−1.77 (0.48) **	−8.66 (2.39) **	−11.75 (2.27) ***	F = 5.69 **	HC > AD = LBD

^‘a’^ indicates that LBD consists of 15 PDD and 18 DLB patients; ^b^ indicates the post-hoc test is Tukey method. The symbols ‘*’,‘**’ and ‘***’ indicates *p* ≤ 0.05, *p* ≤ 0.01 and *p* ≤ 0.001, respectively. As for post-hoc analyses, the symbols ‘<’ and ‘>’ indicates *p* ≤ 0.05 while ‘=’ indicates *p* > 0.05. S.E. means standard errors. The units for RT and accuracy are millisecond (ms) and percentage (%).

**Table 3:** Summary of the EEG source-level results at 1–100 Hz.

Within-Group One Sample T Tests				
Patient Group	Alert	Orient	Conflict	Executive
**LBD**	Temporal_mid_R; Frontal_mid_orb_L/R Frontal_mid_L/R Frontal_sup_L/R; Frontal_inf_orb_R; Frontal_inf_tri_R; Frontal_inf_oper_R; Calcarine_L; Lingual_R; Rolandic_oper_R; Postcentral_L/R Precentral_R;	Temporal_inf_L/R Temporal_mid_L/R Frontal_sup_orb_L/R Frontal_inf_tri_R Calcarine_L/R Occipital_inf_L/R Postcentral_L/R	Temporal_inf_L/R Frontal_sup_orb_L/R Frontal_inf_tri_R Frontal_mid_R Calcarine_L Parietal_sup_R Occipital_mid_R Rolandic_oper_L/R Postcentral_L/R Precentral_R	Temporal_inf_L Temporal_sup_R Frontal_sup_orb_L/R Frontal_inf_orb_R Frontal_inf_Tri_L/R Frontal_mid_L/R Calcarine_L Parietal_sup_L Parietal_inf_L Occipital_mid_L Rolandic_oper_R Postcentral_L/R Precentral_R Insular_R
**AD**	Frontal_inf_oper_R Frontal_mid_L/R Precentral_L/R Postcentral_L/R	Temporal_inf_L/R Frontal_sup_orb_L/R Frontal_inf_orb_R Rolandic_oper_L	Frontal_inf_tri_L/R Frontal_inf_oper_R Frontal_mid_R Precentral_R Insula_L/R	Frontal_sup_orb_L/R Frontal_mid_orb_L/R Front_inf_orb_L/R Front_mid_R Insula_L
**HC**	Frontal_sup_orb_L/R Frontal_inf_tri_R	Temporal_sup_L Temporal_inf_L Frontal_sup_orb_R/L Frontal_inf_orb_R Rolandic_oper_L	Temporal_inf_L/R Frontal_sup_orb_L/R Frontal_mid_orb_L/R	Temporal_inf_L Front_sup_orb_L/R Front_inf_tri_L Front_mid_orb_L/R
**Between-group One Way ANOVA**				
	**Alert**	**Orient**	**Conflict**	**Executive**
**HC > AD &** **HC > LBD**	Frontal_inf_tri_R Frontal_mid_L Frontal_sup_L Parietal_inf_L	Precentral_L Frontal_inf_oper_L	/	Front_inf_oper_L Front_mid_L Precentral_L Thalamus_L/R
**AD > HC &** **AD > LBD**	Frontal_inf_oper_R Precentral_R/L Postcentral_R	/	Frontal_inf_tri_L/R Frontal_inf_oper_R Frontal_mid_R Precentral_R Insula_L/R	Front_inf_Oper_L Insula_L
**LBD > HC**	/	/	/	Insula_R
**LBD > AD**	/	/	/	/

**Table 4:** Summary of impairment network and oscillation effects for AD and LBD patients.

One Sample T Test (1–100 Hz)	Alert	Orient	Conflict	Executive
AD	bilateral middle frontal areas bilateral precentral areas bilateral postcentral areas	right temporal areas	bilateral inferior frontal areas bilateral insular lack of inferior temporal areas (α)	bilateral inferior frontal areas left insular lack of inferior temporal areas (α)
LBD	bilateral middle frontal areas right precentral areas bilateral postcentral areas temporal-to-occipital network	temporal-central-occipital network	right inferior frontal areas central-to-occipital areas	bilateral inferior frontal areas right insular central-to-occipital areas
**Group Comparisons**	**Alert**	**Orient**	**Conflict**	**Executive**
HC > AD & HC > LBD	left superior frontal areas (β γ) right inferior frontal areas (θ α β γ) left inferior parietal areas (β γ)	left inferior frontal areas (α β γ) left precentral areas (α β γ)	/	left inferior frontal areas (α β γ) left precentral areas (null) left middle frontal areas (α β γ) left occipital areas (α β γ)
AD > HC & AD > LBD	bilateral middle frontal areas (θ α β γ) bilateral precentral areas (θ α β γ) right postcentral areas (null) right inferior frontal areas (α β γ)	/	right inferior frontal areas (θ α β γ) left inferior frontal areas (θ α β γ) bilateral insular (θ α β γ) right middle frontal areas (null) right precentral areas (θ α β γ)	left inferior frontal areas (θ α β γ) left insular (null)
LBD > HC	/	/	/	right inferior frontal areas (θ β γ) right insular (θ β γ)

Notes: the null means no significant oscillatory differences were found for the area. The symbols θ α β γ indicate different frequencies: theta band, 4–7 Hz; alpha band, 8–13 Hz; beta band, 14–30 Hz; gamma band, 31–100 Hz.

## Data Availability

All datasets are available upon request.

## Abbreviations

CAMCOG = Cambridge Cognitive Examination; MMSE = Mini-Mental State Examination; UPDRS = Unified Parkinson’s Disease Rating Scale; NPI = Neuropsychiatric Inventory; CAF = Clinician Assessment of Fluctuation; HC = Healthy Controls; AD = Alzheimer’s disease; LBD = Lewy Body Dementia; DLB = Dementia with Lewy Bodies; PDD = Parkinson’s Disease Dementia; ANOVA = Analysis of Variance; RT = Reaction time; ANT = Attention Network Task; FWE = Family Wise Error
